# Cytochrome P450 monooxygenase genes in the wild silkworm, *Bombyx mandarina*

**DOI:** 10.7717/peerj.10818

**Published:** 2021-02-03

**Authors:** Linrong Wan, Anlian Zhou, Wenfu Xiao, Bangxing Zou, Yaming Jiang, Jinshu Xiao, Cao Deng, Youhong Zhang, The members of the Genefang Research Team: Zi-yan Huang, Cong-fan Bu, Jie Zeng, Zhao-nan Hao, Yan-peng Chen, and Meng-jia Liu

**Affiliations:** 1 Sericultural Research Institute, Sichuan Academy of Agricultural Sciences, Nanchong, Sichuan, China; 2 College of Agronomy, Sichuan Agricultural University, Chengdu, Sichuan, China; 3 Research and Development Center, Genefang, Chengdu, Sichuan, China; 4 Departments of Bioinformatics, DNA Stories Bioinformatics Center, Chengdu, Sichuan, China

**Keywords:** *Bombyx mandarina*, Wild silkworm, Cytochrome P450, Gene family

## Abstract

Wild (*Bombyx mandarina)* and domestic silkworms (*B. mori*) are good models for investigating insect domestication, as 5000 years of artificial breeding and selection have resulted in significant differences between *B. mandarina* and *B. mori*. In this study, we improved the genome assemblies to the chromosome level and updated the protein-coding gene annotations for *B. mandarina*. Based on this updated genome, we identified 68 cytochrome P450 genes in *B. mandarina*. The cytochrome P450 repository in *B. mandarina* is smaller than in *B. mori*. Certain currently unknown key genes, rather than gene number, are critical for insecticide resistance in *B. mandarina,* which shows greater resistance to insecticides than *B. mori*. Based on the physical maps of *B. mandarina,* we located 66 cytochrome P450s on 18 different chromosomes, and 27 of the cytochrome P450 genes were concentrated into seven clusters. KEGG enrichment analysis of the P450 genes revealed the involvement of cytochrome P450 genes in hormone biosynthesis. Analyses of the silk gland transcriptome identified candidate cytochrome P450 genes (*CYP306A*) involved in ecdysteroidogenesis and insecticide metabolism in *B. mandarina*.

## Introduction

The domestic silkworm, *Bombyx mori*, is a model insect often used to study physiology, biochemistry, developmental biology, neurobiology, and pathology (Kawamoto et al., 2019). It has been reared for more than 5,000 years for silk production (Fang et al., 2015), and it is now used for commercial production of medically and industrially important biomaterials based on genetic engineering. The wild silkworm (*B. mandarina*), the direct ancestor of *B. mori*, is a valuable gene pool resource that can be exploited and utilized. *B. mandarina* provides an important basic material to study the origin and differentiation of silkworms and is significant for *B. mori* breeding and the establishment and application of a gene pool for special traits.

A draft sequence of the *B. mori* genome was first reported by Chinese and Japanese groups (Xia et al., 2004), and the annotation of both WGS data sets with 8.48x sequence coverage was completed in 2008 (International Silkworm Genome C, 2008). Since then, many transcriptomic and evolutionary studies have investigated important bio-systems differing between the domestic and wild silkworms based on the genome sequence. Kawamoto et al. (2019) performed hybrid assembly of *B. mori* based on 140 × deep sequencing of long (PacBio) and short (Illumina) reads and annotated the new genome with more RNASeq and protein data, generating higher quality genome assemblies and more accurate gene models. To reconstruct the domestication processes and to identify selective sweeps among *B. mori* strains, Xiang et al. (2018) generated a draft assembly for *B. mandarina* using a classic shotgun approach based on Illumina sequencing platforms. These high-throughput datasets provide a more comprehensive way to study the similarities and differences between *B. mori* and *B. mandarina* at the whole genome level.

The cytochrome P450 monooxygenases (P450s) genes are a large and complex gene superfamily of heme-thiolate proteins. They are found in most organisms from prokaryotes to eukaryotes (Nelson, 1999). The cytochrome P450 genes are an ancient enzymatic system, and all of the current cytochrome P450s may have descended from a common ancestral gene (Ai et al., 2011; Yu et al., 2015). P450 genes are responsible for the oxidative metabolism of structurally diverse endogenous and exogenous compounds (Nebert & Gonzalez, 1987). In insects, the cytochrome P450 enzymes catalyze the metabolism of physiologically important endogenous compounds, including hormones and pheromones at juvenile and molting stages, and they are well known for their detoxification in pesticides (Scott, 1999; Scott, Liu & Wen, 1998). For this reason, many cytochrome P450s have been studied in insects, including *Drosophila melanogaster*, *Anopheles gambiae*, *Aedes aegypti*, *Tribolium castaneum*, *Apis mellifera*, and *B. mori*.

Based on the silkworm draft genome, Ai et al. (2011) identified 84 CYP-related sequences, which were classified into 26 families and 47 subfamilies according to the standard nomenclature. However, Kawamoto et al. (2019) identified a total of 83 cytochrome P450 genes using a high-quality genome assembly. Considering the important role of *B. mandarina* for *B. mori* breeding and the important roles of cytochrome P450s in silkworms, it is interesting to explore the cytochrome P450 genes in *B. mandarina* and compare them to those in *B. mori*. In this study, the cytochrome P450 genes in *B. mandarina* were identified and a comprehensive genome-wide comparative analysis of the cytochrome P450 genes in *B. mandarina* and *B. mori* was performed. We linked the *B. mandarina* scaffold sequences generated by Xiang et al. (2018) into pseudo-molecules using their syntenic to chromosomes in the *B. mori*. This study illuminates the functional diversities and the evolutionary mechanisms and significance of cytochrome P450s in both *B. mori* and *B. mandarina*.

## Materials & Methods

### Genome and annotation data resources

Genomic and annotation data for *B. mori* were downloaded from SilkBase (http://silkbase.ab.a.u-tokyo.ac.jp/cgi-bin/download.cgi). This genome was published in 2019 and based on 140 × deep sequencing of long (PacBio) and short (Illumina) reads. The new genome was annotated with more RNASeq and protein data, generating higher quality genome assemblies and more accurate gene models than the previous version (Kawamoto et al., 2019). Genomic and annotation data for *B. mandarina* were downloaded from the NCBI RefSeq (accession: GCF_003987935.1, published in 2019) and assembled using a classic whole genome shotgun approach, based on Illumina sequencing platforms (Xiang et al., 2018). Genome and annotation datasets for other insect species are shown in Table S1.

### Improving the quality of the *B. mandarina* genome assembly

To build chromosomes from genome contigs or scaffolds, we used an alignment-based approach. We used Lastz (Harris, 2007) to perform the whole genome alignment between *B. mandarina* (Xiang et al., 2018) and *B. mori* (Kawamoto et al., 2019). Then, using the syntenic alignments between *B. mandarina* contigs and scaffolds and *B. mori* chromosomes, we recorded the strands and turns of *B. mandarina* contigs and scaffolds relative to the *B. mori* chromosomes. Finally, the wild and domestic contigs and scaffolds were linked into pseudo-molecules according to the strands and turns information (Table S2).

### Updating the gene annotation of *B. mandarina*

The original *B. mandarina* NCBI RefSeq protein-coding gene models were generated by the automated NCBI Eukaryotic Genome Annotation Pipeline (https://www.ncbi.nlm.nih.gov/genome/annotation_euk/Bombyx_mandarina/100/). We first removed the low-quality protein-coding genes with coding sequences (CDS) shorter than 150 bp and with stop codons in the CDSs. For the protein-coding gene models with alternative splicing, we only kept the longest CDS for each gene to generate a clean RefSeq annotation file in GFF3 format.

Transcriptome sequencing raw data were downloaded from previous studies (Table S3). To obtain high-quality clean reads, the raw sequencing reads were filtered using Trimmomatic software (Bolger, Lohse & Usadel, 2014) with the following steps. First, reads with adaptor sequences were removed. Then, reads containing more than 20% of low-quality bases (Q <  20) or containing more than 3% of ambiguous ‘N’ were discarded. The reads were also trimmed where the four-bases-window had an average quality lower than 20. After these filtering steps, the clean reads from each sample were aligned to the updated genome assemblies using HiSAT2 (version 2.0.4) (Kim, Langmead & Salzberg, 2015), which generated BAM files for downstream analyses.

The transcripts in each sample were assembled and merged using StringTie (version 1.3.1c) (Pertea et al., 2015) and the BAM files were generated by the HiSAT2 (Kim, Langmead & Salzberg, 2015). If none of the transcripts in a gene model had overlaps with the RefSeq protein-coding gene models, the transcripts in this gene were subjected to TransDecoder (version r20140704) (Haas, 2014) to predict the potential coding sequences (CDSs). This gene model was defined as a novel protein-coding gene model if it met the following requirements: (1) the CDSs had at least 50 codons; and (2) the CDSs had start- and stop-codons. We only retained the longest CDS for each novel protein-coding gene model.

To determine the functional annotation of the protein-coding gene models, a BLASTP (Camacho et al., 2009) search with an *E*-value ≤ 1e^−5^ was performed against protein databases, including NR (non-redundant protein sequences in NCBI) and SwissProt (Boeckmann et al., 2003). KEGG annotation that maps the *B. mandarina* cytochrome P450 genes to possible KO numbers and map numbers was fetched from KOBAS (version 3.0) (Xie et al., 2011) results. The domains and GO terms of each gene model were predicted by InterProScan (Quevillon et al., 2005) against public protein databases, including ProDom (Bru et al., 2005), PRINTS (Attwood et al., 1994), Pfam (Bateman et al., 2004), SMART (Ponting et al., 1999), PANTHER (Mi et al., 2005), PROSITE (Hulo et al., 2006), and TIGR (Haft, Selengut & White, 2003).

### Synteny analysis of *B. mandarina* and *B. mori*

To explore the collinearity of *B. mandarina* and *B.mori*, the updated *B. mandarina* proteome was blasted against to the *B. mori* proteome, and the MCScanX (Wang et al., 2012) was used to detect syntenic genes and blocks (regions with at least eight collinear genes). The collinearity of genes between *B. mandarina* and *B. mori* was visualized using Circos (Krzywinski et al., 2009).

### In silico identification of cytochrome P450 genes in *B. mandarina* and other insects

The cytochrome P450 genes of *B. mandarina* were identified as following steps. First, we collected total 84 cytochrome P450 genes in *B. mori*, which included 82 cytochrome P450 genes from Kawamoto et al. (2019) and another two cytochrome P450 genes identified by Ai et al. but missing in Kawamoto et al. (Ai et al., 2011). The proteins and CDSs were extracted for downstream analyses. We also downloaded 91 P450 proteins from *Drosophila melanogaster* (http://drnelson.uthsc.edu/cytochromeP450.html), and then the cytochrome P450 proteins from *D. melanogaster* and *B. mor* i were combined to form a reference cytochrome P450 protein database. Second, the *B. mandarina* proteome was searched against the reference cytochrome P450 protein database using the BLASTP algorithm (Camacho et al., 2009), with an e-value cut-off of 1e−5. For each *B. mandarina* protein, the top hit to the reference cytochrome P450 protein database was remained, and the *B. mandarina* proteins with hits to the reference cytochrome P450 proteins were the candidate cytochrome P450 proteins. Third, the domains of the *B. mandarina* cytochrome P450 candidates were annotated by the iprscan (version 5) (Quevillon et al., 2005) using the Pfam database (http://pfam.sanger.ac.uk/) (Bateman et al., 2004), and the candidate was identified as the cytochrome P450 gene if it had the PF00067 domain (cytochrome P450). Additional TBLASTN (Camacho et al., 2009)searches against the *B. mandarina* genomic assemblies followed by gene structure refinement using GeneWise (Birney, Clamp & Durbin, 2004) were also performed with the reference cytochrome P450 protein database to avoid missing cytochrome P450-related genes. We further manually corrected annotation for three genes (XP_028036989.1, XP_028036546.1 and XP_028040841.1) that were mis-predicted to generate artificial fusion genes by the NCBI Eukaryotic Genome Annotation Pipeline, and for these loci, we applied these criteria to define genuine protein-coding genes: (1) insertions, deletions or frameshifts were not allowed when they were compared to their homologous protein; (2) start codon and stop codon were compulsive, and (3) they had at least 50 amino acids.

The cytochrome P450 genes in other insect species (Table S1) were identified using the same pipeline. The exons and domains positions of silkworm cytochrome P450 genes were extracted from GFF3 file and Iprscan results, respectively, and then were plotted using the iTOL (interactive Tree Of Life, https://itol.embl.de/) (Letunic & Bork, 2019). The chromosomal locations for cytochrome P450 genes were plotted using karyoploteR (Gel & Serra, 2017).

### Phylogenetic analyses and classification of *B. mandarina* cytochrome P450 genes

The cytochrome P450 proteins were aligned using MUSCLE v3.8.31 (Edgar, 2004) with default parameters. RAxML (version 8.2.7) (Stamatakis, 2014) was used to generate maximum likelihood with PROTGAMMALGX model and 100 bootstraps. Trees were plotted by the iTOL (https://itol.embl.de/) (Letunic & Bork, 2019). The phylogenetic tree between *B. mandarina* and *B. mori* and the standard nomenclature of *B. mori* were used to classify and name the *B. mandarina* cytochrome P450 genes (Ai et al., 2011; Kawamoto et al., 2019).

### RNA-Seq expression analysis of cytochrome P450 genes

The gene expression levels of each sample were quantified using HTSeq (version 0.9.1) (Anders, Pyl & Huber, 2014) and the BAM files generated by the HiSAT2 (Kim, Langmead & Salzberg, 2015). Then, the DEGs between different sample pairs were detected via the edgeR software package (Robinson, McCarthy & Smyth, 2010) of the R language. The *P*-values were corrected for false discovery rate (FDR) using multiple tests. Differentially expressed genes (DEGs) met the following criteria: FDR (adjusted P) <0.05 and —log2 fold change (FC)—>0.5.

### GO and KEGG enrichment analysis

Significantly over-represented GO terms among the *B. mandarina* cytochrome P450 genes were identified using the topGO package (Alexa & Rahnenfuhrer, 2010) in R programming language ( https://www.r-project.org/). The enrichment of KEGG pathways was conducted with Fisher’s exact test using R scripts. The significantly over-represented GO terms and KEGG maps were identified with adjusted *p*-values ≤ 0.05.

## Results

### Improvement of genome assembly to the chromosome level

**Figure 1:**
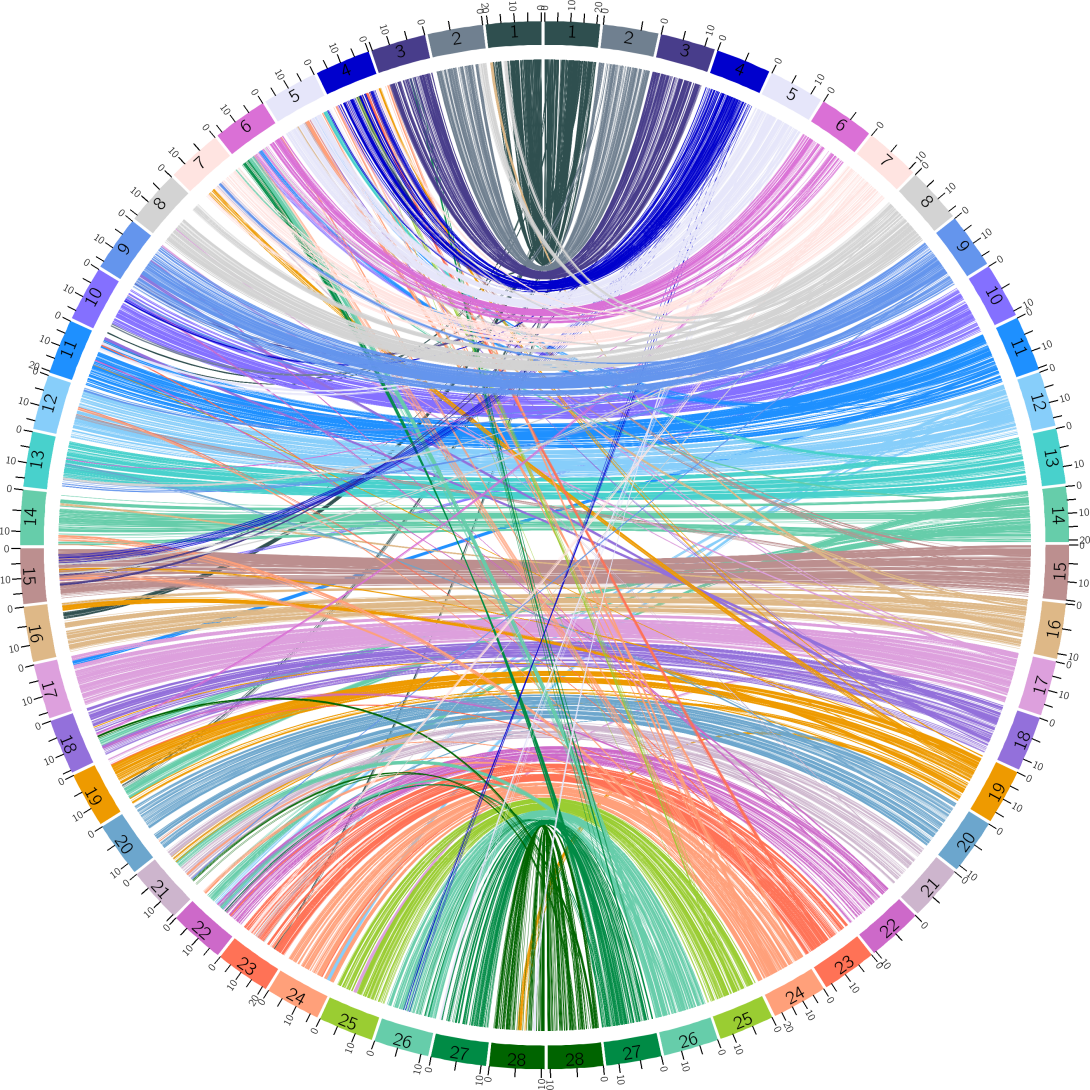
Synteny between *B. mandarina* and *B. mori*. The left 28 chromosomes are from the *B. mandarina* genome and the right 28 chromosomes are from the *B. mori* genome. Echo chromosome number is marked on the karyotype bar on the circle, and ticks on each bar are the physical positions (unit: Mbp). Links are the homologous gene pairs identified by MCScanX and colored using the *B. mori* chromosome color scheme.

The draft assembly for *B. mandarina* described by Xiang et al. was fragmental and low quality (Xiang et al., 2018). There are a total of 3,105 scaffolds with a total length of 398,588,931 bp (395,983,407 bp without gaps). The scaffold and contig N50 were 2,789,315 bp and 29,637 bp, respectively. The minimum sequence length was 500 bp, and the maximum sequence length was 14,129,094 bp. To improve the quality of genome assembly for *B. mandarina*, we implemented a reference-assisted approach to build chromosomes from genome contigs or scaffolds published by Xiang et al. (2018). A total of 337 scaffolds were linked into 28 chromosomes, and the total length of these chromosomes reached 98.35% (392,034,257 bp) of the raw assemblies published by Xiang et al. *.* (Xiang et al., 2018) (Table S2). The shortest chromosome was chr2 (7,258,460 bp), while the longest one was chr24 (22,989,029 bp). Chr12 had the smallest size variation among B. mandarina and *B. mori*, while chromosome 4 had the largest size variation. To study the synteny of the two silkworms, the collinearity of genes between *B. mandarina* and *B. mori* was visualized using Circos (Fig. 1).

### Identification of novel protein-coding genes and gene function annotation

To obtain more protein-coding gene models in the *B. mandarina* genome, we updated the gene annotation using StringTie-TransDecoder pipeline. Using these NCBI RefSeq representative protein-coding gene models as a reference, the StringTie assembled a total of 26,061 gene models with 41,390 transcripts. Among them, 14,005 gene models containing 29,053 novel transcripts (53.74% of total gene models) had no overlaps with the NCBI RefSeq representative protein-coding gene models, and were defined as candidate novel genes. The candidate novel gene sequences were scanned directly for CDSs with TransDecoder (Haas, 2014), which generated 1,939 novel protein-coding gene models after filtering out low-quality CDSs (see filtering criteria in the Methods). Together with a manually annotated cytochrome P450 gene (see next section), we identified 1,940 novel protein-coding gene models in the *B. mandarina* genome. When incorporating the NCBI RefSeq annotation, there were 14,212 protein-coding gene models for the *B. mandarina* genome.

Among these 1,940 novel protein-coding genes, 1,295 genes (66.75%) could be functionally annotated to at least one of the four databases, including NR (non-redundant protein sequences in NCBI), SwissProt (Boeckmann et al., 2003), KEGG, and GO. For the whole *B. mandarina* proteome, approximately 94.38% of genes could be functionally annotated compared with the 95.14% in *B. mori*.

### The identification and classification of cytochrome P450 genes in *B. mandarina*

By integrating the results from homologous to *B. mori* cytochrome P450 proteins and the existence of complete cytochrome P450 domain (PF00067), we identified 67 cytochrome P450 genes in the *B. mandarina* genome. To find all of the cytochrome P450-related genes in the genomes, we further manually annotated the genome using the *B. mori* cytochrome P450 proteins as a reference (see Methods section). This homology-based gene annotation strategy detected one additional P450 gene, resulting in a total of 68 cytochrome P450 genes in the *B. mandarina* genome. For *B. mori*, Ai et al. (2011) identified 84 cytochrome P450 sequences based on a silkworm draft genome, while Kawamoto et al. (2019) identified 83 cytochrome P450 genes with a high-quality genome assembly and a unified set containing 84 cytochrome P450 genes was used in our downstream analyses (Table 1).

**Table 1:** Cytochrome P450 genes in *B. mandarina* and *B. mori*. For *B. mori*, genes marked with ‘*’ and ‘$’ have two counterparts in *B. mandarina*, and genes with ‘M’ mean manually modified by Kawamoto et al. (2019). For *B. mandarina*, genes with bold font involves in the KEGG map00981 (Insect hormone biosynthesis), and their amino acid (AA) length, exon number and protein sequence identity to *B. mori* homologs are listed in the last three column.

*B. mandarina*	*B. mori*	Gene	AA length	Exon number	Identity (%)
*CYP2Clan*					
**XP_028039023.1**	KWMTBOMO06658	CYP15C1	390	8	98.71
**XP_028040961.1**	KWMTBOMO05795	CYP18A1	541	7	100.00
**XP_028040963.1**	KWMTBOMO05796	CYP18B1	533	7	99.15
XP_028034668.1	KWMTBOMO00033	CYP303A1	498	3	99.00
MSTRG.449.1	KWMTBOMO00224	CYP305B1	487	9	100.00
**XP_028040956.1**	KWMTBOMO05794	CYP306A1	538	8	100.00
**XP_028041590.1**	KWMTBOMO06147	CYP307A1	536	2	99.51
*CYP3Clan*					
XP_028040226.1	KWMTBOMO12795	CYP324A1	499	9	99.60
XP_028035792.1	KWMTBOMO08858	CYP332A1	500	8	99.58
XP_028039903.1	KWMTBOMO09327	CYP337A2	488	2	98.98
XP_028039890.1	KWMTBOMO09329	CYP337A1	489	2	98.57
XP_028030266.1	KWMTBOMO07090	CYP338A1	464	2	98.28
XP_028037108.1	KWMTBOMO04531	CYP354A1	516	9	99.03
–	KWMTBOMO12722	CYP365A1	–	–	–
XP_028030488.1	KWMTBOMO06852	CYP6AB5	513	2	98.44
MSTRG.11650.2	KWMTBOMO12342	CYP6AB4	521	2	91.77
–	KWMTBOMO12343	CYP6AB8	–	–	–
XP_028037754.1	KWMTBOMO07237	CYP6AE9	517	2	97.87
XP_028026771.1	KWMTBOMO09944*	CYP6AE7	515	2	97.67
XP_028026792.1	KWMTBOMO09944*	CYP6AE7	523	2	85.24
XP_028026744.1	KWMTBOMO09945	CYP6AE6P	515	4	100.00
–	KWMTBOMO09947	CYP6AE5	–	–	–
–	KWMTBOMO09950	CYP6AE5	–	–	–
XP_028026755.1	KWMTBOMO09951-1M	CYP6AE4	523	2	94.22
XP_028026857.1	KWMTBOMO09951-2M	CYP6AE2	523	2	99.04
XP_028026790.1	KWMTBOMO09952$	CYP6AE3P	523	2	96.56
XP_028026883.1	KWMTBOMO09952$	CYP6AE3P	523	2	96.56
XP_028033575.1	KWMTBOMO13805	CYP6AE22	516	2	99.81
MSTRG.12374.1	KWMTBOMO13412	CYP6AN2	515	3	99.21
XP_028037243.1	KWMTBOMO12654	CYP6AU1	496	2	99.80
CYP6AV1-Bman	CYP6AV1	CYP6AV1	500	2	99.800
–	KWMTBOMO05640	CYP6AW1	–	–	–
XP_028027733.1	KWMTBOMO12622	CYP6B29	505	2	100.00
XP_028036989.1	KWMTBOMO10620	CYP9A20	531	10	98.87
XP_028036963.1	KWMTBOMO10621-1M	CYP9A19	490	10	90.67
XP_028042624.1	KWMTBOMO10621-2M	CYP9A21	158	3	83.70
XP_028036964.1	KWMTBOMO10626	CYP9A22	531	11	98.87
XP_028033856.1	KWMTBOMO10600	CYP9AJ1	505	10	98.01
XP_028035894.1	KWMTBOMO09346	CYP9G1	495	9	92.53
XP_028036982.1	KWMTBOMO10603	CYP9G3	525	10	99.43
*CYP4Clan*					
–	KWMTBOMO15697	CYP340A1	–	–	–
–	KWMTBOMO15698	CYP340A5P	–	–	–
XP_028040572.1	KWMTBOMO15699	CYP340A4	488	10	97.10
XP_028040567.1	KWMTBOMO15700	CYP340A2	460	9	95.38
–	KWMTBOMO15704-15705M	CYP340A6	–	–	–
XP_028040566.1	KWMTBOMO15707	CYP340A3	489	10	99.80
XP_028043542.1	KWMTBOMO15837	CYP340B1	484	10	96.23
XP_028043540.1	KWMTBOMO15838	CYP340C1	495	11	99.02
XP_028040570.1	KWMTBOMO15694	CYP340D1	487	10	100.00
–	KWMTBOMO15695	CYP340E1	–	–	–
XP_028031815.1	KWMTBOMO15685	CYP340F1	491	10	98.45
XP_028043541.1	KWMTBOMO15835	CYP340un1	487	12	97.94
–	CYP341A6	CYP341A6	–	–	–
XP_028036361.1	KWMTBOMO13274-13275M	CYP341A1	508	10	98.23
XP_028036360.1	KWMTBOMO13276	CYP341A3	504	10	94.82
XP_028036359.1	KWMTBOMO13278	CYP341A4	508	10	85.32
XP_028036358.1	KWMTBOMO13279	CYP341A5	508	10	99.16
XP_028036416.1	KWMTBOMO13280	CYP341A7	379	8	98.33
XP_028040208.1	KWMTBOMO13324	CYP341B1	512	10	99.34
XP_028026371.1	KWMTBOMO13451	CYP341C1	505	10	99.51
MSTRG.17906.1	KWMTBOMO01080	CYP366A1	557	11	99.55
–	KWMTBOMO09792	CYP367A1	–	–	–
XP_028036546.1	KWMTBOMO09791	CYP367B1	497	10	96.58
XP_028041506.1	KWMTBOMO01330	CYP4AU2	495	10	99.09
–	KWMTBOMO12747	CYP4AX1	–	–	–
–	KWMTBOMO12748	CYP4AX2	–	–	–
XP_028042103.1	KWMTBOMO07943	CYP4G22	556	9	100.00
XP_028033176.1	KWMTBOMO07978	CYP4G24	562	11	94.61
XP_028033210.1	KWMTBOMO07979-1M	CYP4G23	562	10	99.47
–	KWMTBOMO07979-2M	CYP4G23	–	–	–
XP_028028458.1	KWMTBOMO07690	CYP4L6	499	11	98.33
–	KWMTBOMO02817	CYP4M9	–	–	–
XP_028040841.1	KWMTBOMO02818	CYP4M5	503	9	99.80
XP_028042717.1	KWMTBOMO12746	CYP4S5	125	1	100.00
–	KWMTBOMO12749	CYP4S6	–	–	–
*Mito.Clan*					
XP_028034514.1	KWMTBOMO08259	CYP301A1	528	8	100.00
**XP_028032374.1**	KWMTBOMO13168	CYP302A1	517	8	98.45
**XP_028030999.1**	KWMTBOMO03959-03960M	CYP314A1	516	9	92.49
XP_028033300.1	KWMTBOMO04611	CYP315A1	496	8	99.58
XP_028042905.1	KWMTBOMO04516	CYP333A2	420	9	98.81
XP_028028513.1	KWMTBOMO07693	CYP333B2	510	10	99.22
XP_028028461.1	KWMTBOMO07694	CYP333B1	512	10	99.41
–	KWMTBOMO04339	CYP333un1	–	–	–
XP_028043257.1	KWMTBOMO11023-11024M	CYP339A1	577	10	99.67
XP_028042777.1	KWMTBOMO08262	CYP49A1	424	10	99.37
–	KWMTBOMO11585	CYP49A2	–	–	–

Although *B. mandarina* has a significantly smaller cytochrome P450 repository than *B. mori*, we did find two duplicated genes in the *B. mandarina*. These genes are all belong to CYP6AE subfamily members. Interestingly, in the cotton bollworm *Helicoverpa armigera*, CYP6AE gene cluster knockout using the CRISPR-Cas9-based genome editing tools reveals their roles in detoxification of phytochemicals and insecticides (Wang et al., 2018). Therefore, the duplicated CYP6AE subfamily members in the *B. mandarina* may contribute to their reduced susceptibility to the insecticides used for control (Bing et al., 2010).

**Figure 2:**
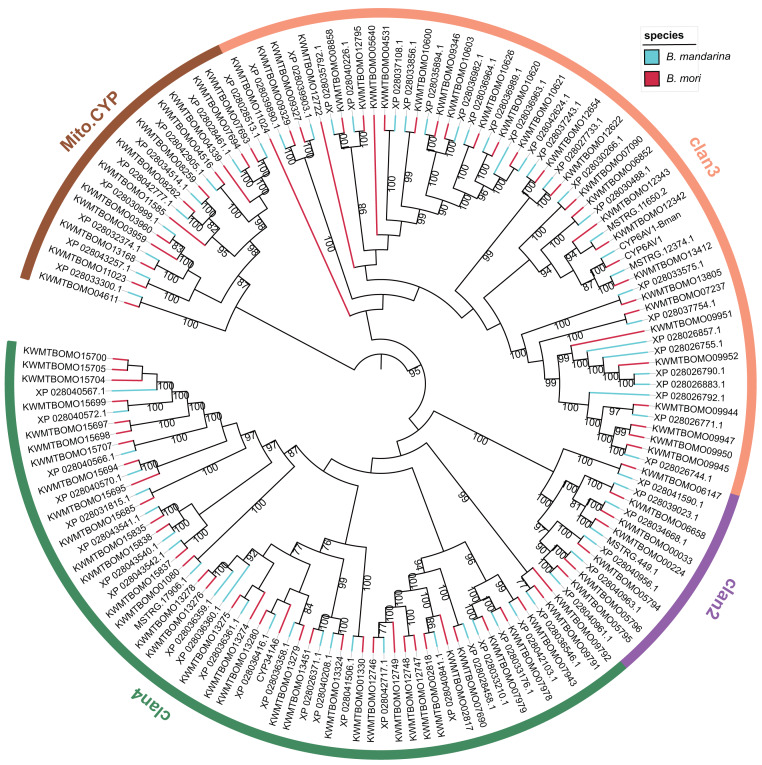
Phylogenetic tree of the cytochrome P450 genes in the wild and domestic silkworms. The bootstrap value for 100 trials is labeled on each branch (only values ≥ 75 are shown). The families attributed to insect P450 genes are marked for each clan on the circle.

We constructed a maximum likelihood tree using the P450 proteins from *B. mandarina* and *B. mori* (Fig. 2). Consistent with previous results, these P450 genes can be grouped into four major clades, which are common in the insects and include the CYP2, CYP3, CYP4 and mitochondrial CYP clades. Using the phylogenetic tree, the 68 P450 genes in *B. mandarina* were classified into 25 families and 45 subfamilies according to the standard nomenclature and classification of P450 genes in *B. mori*. When compared with *B. mori,* the CYP365 family is missing in *B. mandarina*. For the two cytochrome P450 genes in *B. mori* identified by Ai et al. but missing in the Kawamoto et al. (Ai et al., 2011), the CYP6AV1 is present in *B. mandarina*, but the CYP341A6 is missing.

The structural divergence of gene family members may arise due to exon/intron loss or gain and other mechanisms, and analyses of exon/intron structures can be important in revealing the evolutionary history of the gene family. The investigation of the intron–exon organizations of *B. mandarina* cytochrome P450 genes revealed highly variable intron-exon structures among these genes (Fig. S1). However, the genes in a clade of the CYP3 clan, which is the largest clan and is most closely related to vertebrate CYP3 and CYP5 families, have significantly smaller numbers of exons and a longer length for each exon than those in the other clans. These similarities in exon−intron organization in this clade provide a strong support for a common origin.

### Genomic distribution of P450 superfamily in the silkworm

Based on the physical maps, we located 66 cytochrome P450s on 18 different chromosomes in *B. mandarina*. Only two genes were on the scaffolds. The XP_028042624.1 (CYP9A21) was on the scaffold NW_021012135.1, while its *B. mori* ortholog was on chromosome 17. The XP_028042717.1 (CYP4S5) was on scaffold NW_021013128.1, while its ortholog in the *B. mori* was on chromosome 21.

**Figure 3:**
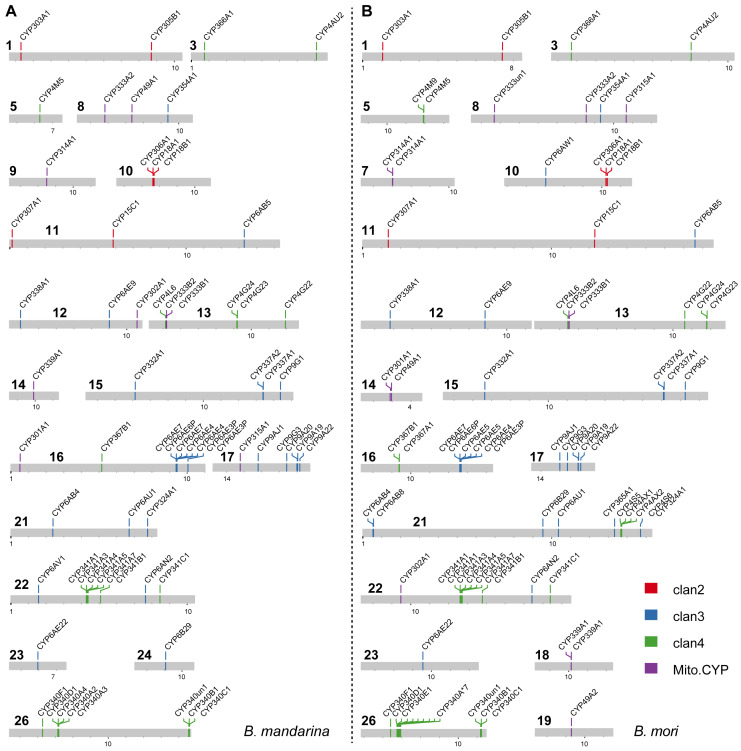
Physical locations of *B. mandarina* (A) and *B. mori* (B) P450 genes on the chromosomes. Chromosomes are grey rectangles, bold number marked on each chromosome is their chromosome number, and ticks on each chromosome are the physical positions (unit: Mbp). P450 genes are colored according their clan group information.

The genomic distribution patterns of the cytochrome P450 genes in these two silkworm species are different. For *B. mandarina*, 27 cytochrome P450 genes were concentrated into 7 clusters, which are defined as containing at least three genes, while for *B. mori*, 37 cytochrome P450 genes were concentrated into 8 clusters (Fig. 3). *B. mori* has one more cluster, which is located on chromosome 21 and has four genes. Three of the four genes were not found in *B. mandarina*, indicating potential loss. The largest single cluster in *B. mori* is located on chromosome 26, which has 9 CYP340 genes; however, the synteny regions in *B. mandarina* only have 4 CYP340 genes.

Figure 3 shows that these two species also share common characteristics in the genomic distribution of cytochrome P450 genes. The P450s are unevenly distributed in the genome. Most of the cytochrome P450s are tandemly arranged on chromosomes in both silkworm species. All of the CYP340 genes are located on chromosome 26 and form at least two clusters. All of the CYP341 genes are located on chromosome 22, and form a cluster and a singleton. CYP9 genes are located in the chromosome 17 and form a cluster, with the exception of *CYP9G1*, which is located on chromosome 15 as a singleton.

### Computational functional analyses of *B. mandarina* cytochrome P450 genes

To explore the functional roles of the cytochrome P450 genes in *B. mandarina*, we obtained the GO terms from Gene Ontology (GO), which classifies genes into three GO categories: cellular component, molecular function, and biological process. Using the topGO (Alexa & Rahnenfuhrer, 2010) package in R programming language (https://www.r-project.org/), we identified four over-represented GO terms for molecular function and one over-represented term for biological process (Table S6). For the molecular function, there were 67 of 107 cytochrome P450 genes annotated to the GO:0020037 (heme binding, adjusted *p*-value: 0) and 3 of 16 cytochrome P450 genes annotated to the GO:0004497 (monooxygenase activity, adjusted *p*-value = 0.045; Table S6). These results are consistent with the fact that the cytochrome P450 genes are heme-containing monooxygenases.

**Figure 4:**
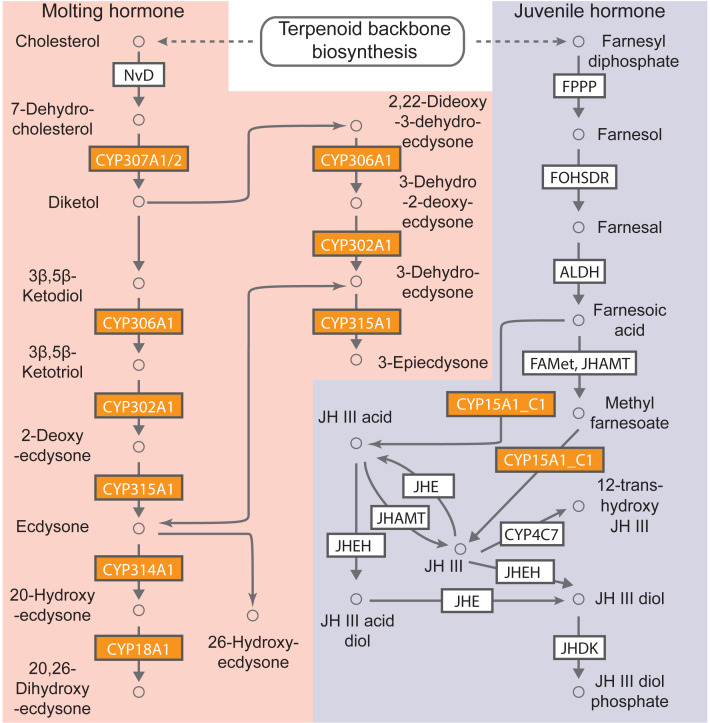
*B. mandarina* cytochrome P450 genes were significantly enriched in the insect hormone biosynthesis pathway. This diagram of insect hormone biosynthesis pathway is adapted from KEGG (map00981), and the boxes colored with yolk yellow are the cytochrome P450 genes found in *B. mandarina*. FPPP, farnesyl diphosphate phosphatase; FOHSDR, NADP+-dependent farnesol dehydrogenase; ALDH, aldehyde dehydrogenase (NAD+); FAMeT, farsoic acid methyltransferase; JHAMT, juvenile hormone-III synthase; JHE, juvenile-hormone esterase; JHEH, juvenile hormone epoxide hydrolase; JHDK, juvenile hormone diol kinase; NvD, cholesterol 7-desaturase.

KEGG pathway-based analysis was also performed to determine the biological functions of cytochrome P450 genes in *B. mandarina*. A total of 63 cytochrome P450 genes (92.65% of total cytochrome P450 genes) could be assigned to 13 non-redundant KO numbers using the KOBAS (Table S7). Among these KO orthologs, the five most abundant KOs were K14999 (gene number: 20, cytochrome P450 family 6 [EC:1.14.-.-]) and K15001 (gene number: 19, cytochrome P450 family 4 [EC:1.14.-.-]). Using KEGG mapper, 7 of these 13 KOs were mapped to the map00981 (Insect hormone biosynthesis), and in our KEGG enrichment analysis, this pathway was also significantly enriched with an adjusted *p* value of 4.48e−18 (Fig. 4). These results revealed the involvement of cytochrome P450 genes in insect hormone biosynthesis.

### Expression pattern of cytochrome P450 genes in silkworms

**Figure 5:**
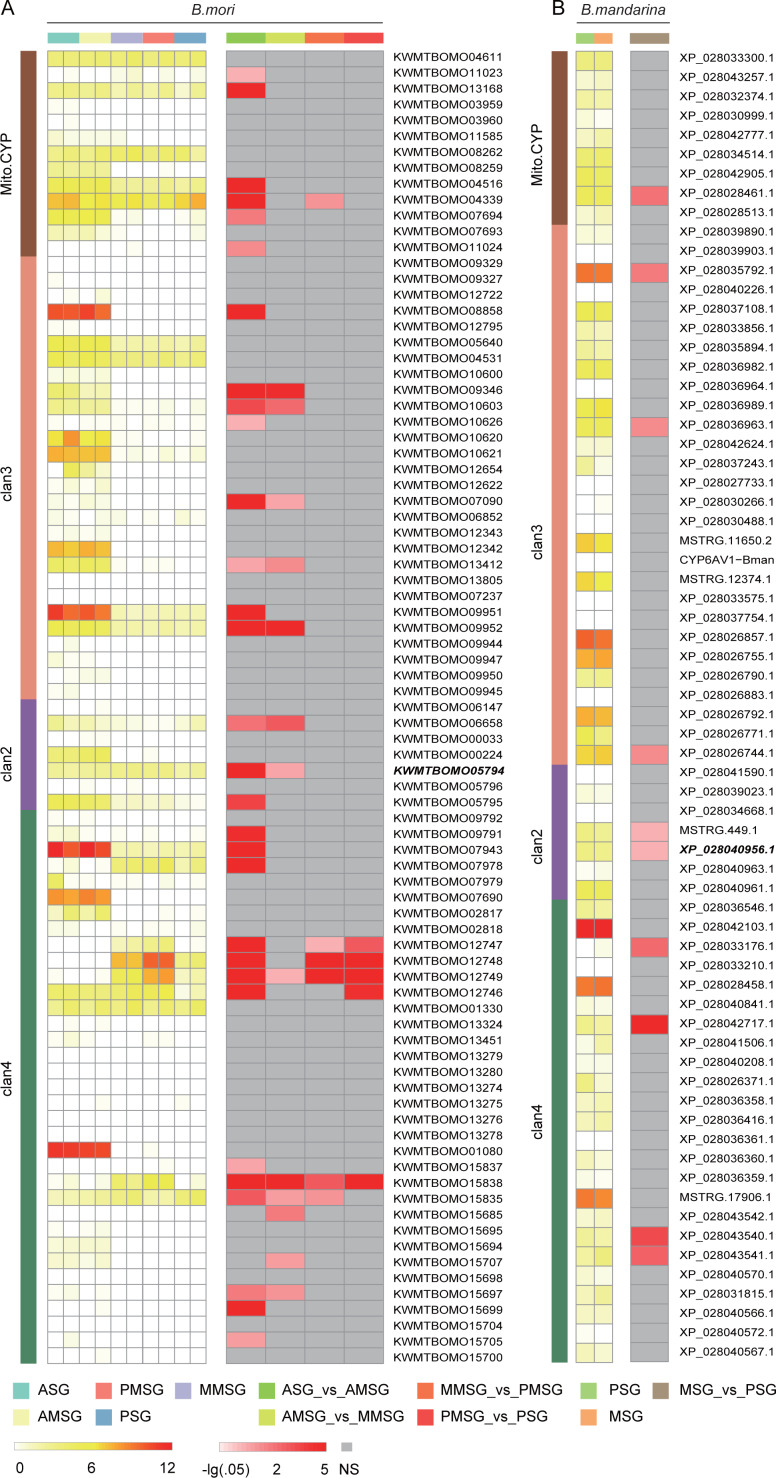
Expression analysis of cytochrome P450 genes across different tissues in *B. mori* (A) and *B. mandarina* (B). The first column is the log10-transformed TMM (trimmed mean of M-values)-normalized expression data, and the tissue types are marked at the top of the heatmap. The second column is the –log10-transformed false discovery rate (FDR) value of DEG analyses from edgeR, and the comparaison scheme are also marked at the top of the heatmap. NS: not significant, namely, FDR > 0.05. Gene ID in Bold style is the *CYP306A1*.

To explore cytochrome P450 gene expression in the silk gland, we collected two RNASeq datasets from silk gland, including the anterior silk gland (ASG), anterior median silk gland (AMSG), middle MSG (MMSG), posterior MSG (PMSG) and posterior silk gland (PSG) (Chang et al., 2015; Cheng et al., 2015). A total of 43 (63% of 68) and 76 (90% of 84) cytochrome P450 genes were expressed (raw counts ≥ 2) in at least one tissue of *B. mandarina* and *B. mori,* respectively. Totally, we identified 10 differentially expressed genes (DEGs) and 32 DEGs in *B. mandarina* and *B. mori* tissues respectively (Fig. 5).

## Discussion

By implementing a reference-assisted approach, we built chromosomes from genome contigs or scaffolds published by Xiang et al. (2018) for *B. mandarina*. The results revealed high genome synteny but also abundant structural rearrangements among the silkworms (Fig. 1). Despite building from a straight-forward alignment-based approach, our reference-assisted chromosome level assemblies can be used in downstream comparative genomic analysis and other types of analyses. We also updated the gene annotation using StringTie-TransDecoder pipeline, and this pipeline identified 1,940 novel protein-coding gene models in the *B. mandarina* genome, indicating the necessity of updating the gene annotation.

We identified 67 cytochrome P450 genes in the *B. mandarina* genome (Table 1). The genomic distribution patterns of the cytochrome P450 genes in these two silkworm species are different (Fig. 3). Computational functional analyses of *B. mandarina* cytochrome P450 genes revealed the involvement of cytochrome P450 genes in insect hormone biosynthesis (Fig. 4). The P450 repository in *B. mandarina* is smaller than in *B. mori* and other insects, except for *Vanessa tameamea* (Table S5 ). This suggests loss of cytochrome P450 genes during evolution. Although *B. mandarina* is very similar to *B. mori* in physiological and morphological characteristics, resistance to insecticides differs between the two species due to natural selection. *B. mori* are weakly resistant to insecticides, and silk production is reduced by >30% annually in China because of insecticide poisoning. In contrast, *B. mandarina* is a major pest of mulberry and are showing reduced susceptibility to the insecticides used for control (Bing et al., 2010). Cytochrome P450 genes play a major role in insecticide resistance, allowing faster metabolic removal of insecticides (Feyereisen, 2015). However, the number of P450 genes in *B. mandarina* is much lower than that in *B. mori*, suggesting that selected key genes, rather than the total gene number of cytochrome P450s, are related to the increased resistance to insecticide resistance in *B. mandarina*.

Silk production is very different in *B. mandarina* and *B. mori* (Chang et al., 2015), and this phenotype attracted many attentions and accumulated many sequencing datasets (Chang et al., 2015; Cheng et al., 2015). The cytochrome P450 enzymes are found in almost all insect tissues. They fulfill many important tasks, from the synthesis and degradation of ecdysteroids and JHs to insecticide metabolism (Feyereisen, 1999). Therefore, it is important to study the expression of cytochrome P450 genes in the silk glands of *B. mandarina* and *B. mori.* The silk gland is the only organ that produces silk proteins (fibroins and sericins). The silk gland is divided into three main parts: anterior silk gland (ASG), median silk gland (MSG), and posterior silk gland (PSG). The MSG can be further divided into anterior, middle, and posterior MSG (AMSG, MMSG, and PMSG, respectively) (Chang et al., 2015). PSGs are responsible for the synthesis and secretion of fibroins, MSG synthesize the sericins (glue proteins), and the ASG processes the liquid silk proteins and secretes them during cocoon formation (Fang et al., 2015). Two RNASeq datasets from the above tissues (Chang et al., 2015; Cheng et al., 2015) were used to explore cytochrome P450 gene expression in the silk gland.

The silk gland is greatly affected by insect hormones, especially, ecdysone and juvenile hormone (JH). Growth and differentiations of the silk gland cells are accelerated by ecdysone, and are controlled by JH (Akai, 1979). Daimon et al. revealed the essential role of CYP15C1 for the JH biosynthesis, and found that this gene is specifically expressed in the corpus allatum, an endocrine organ that synthesizes and secretes JHs (Daimon et al., 2012). However, we found that this gene is also expressed in the silk gland, although at a very low expression level (Fig. 5). Cheng Dao-Jun et al. (2014) found four cytochrome P450 genes involved in ecdysteroidogenesis, including *CYP306A1*, *CYP302A1*, *CYP315A1*, and *CYP314A1*. Among these four genes, the *CYP306A1* was found significantly differently expressed in both *B. mandarina* (XP_028040956.1) and *B. mori* (KWMTBOMO05794). *CYP302A1* was only found to be significantly differently expressed in *B. mori* (KWMTBOMO13168) but not in *B. mandarina* (XP_028032374.1), while the *CYP315A1* (KWMTBOMO04611 for *B. mori*, XP_028033300.1 for *B. mandarina*), and *CYP314A1* (KWMTBOMO03959-03960M for *B. mori,* XP_028030999.1 for *B. mandarina*) were not differently expressed in the two silkworms (Fig. 5).

Phoxim exposure is toxic to silkworms, causes a decrease of fibroin synthesis, and affects silk production. After phoxim exposure, Cheng et al. (2018) found that the transcriptional levels of *CYP6AB* and *CYP306A* were up-regulated by 1.731- and 1.221-fold, respectively. There are two and three *CYP6AB* genes in *B. mandarina* (XP_028030488.1, MSTRG.11650.2) and *B. mori* (KWMTBOMO06852, KWMTBOMO12342, and KWMTBOMO12343), respectively; however, they are not significantly expressed. Interestingly, *CYP306A* was found to be significantly differently expressed in both *B. mandarina* (XP_028040956.1) and *B. mori* (KWMTBOMO05794) (Fig. 5). We also checked the expression of *CYP306A* in the midgut from the *B. mandarina*, which tissues is one of the major tissues for insecticides metabolization. We blasted the *CYP306A* sequences to the RNASeq assemblies in the SilkBase (Kawamoto, 2017), and the expression was validated by its homologous sequences in the SilkBase (A_BomaMG_comp25068_c0_seq1 and A_BomaMG_comp25068_c0_seq2).

## Conclusions

In this study, we improved the quality of the genome assemblies and updated the protein-coding gene annotations for *B. mandarina* using the genome of *B. mori* as reference. Using *in silico* analyses of *B. mandarina* genomes, we identified 68 cytochrome P450 genes. Comparison with other insects revealed that *B. mandarina* may have lost many cytochrome P450 genes. Analyses of the silk gland transcriptome identified candidate cytochrome P450 genes (such as *CYP306A*) involved in ecdysteroidogenesis and insecticide metabolism in *B. mandarina*. Altogether, these results provided a genome-wide glimpse of the *B. mandarina* cytochrome P450 repository; however, the up- or down-regulated cytochrome P450 genes require more wet experiments to explore their biological roles.

##  Supplemental Information

10.7717/peerj.10818/supp-1 Supplemental Information 1 Supplemental tables and figures.

## Additional Information and Declarations

### Data Availability

The following information was supplied regarding data availability: The raw measurements are available as a Supplementary File.
