# Supplementary material for: Cytochrome P450 monooxygenase genes in the wild silkworm, Bombyx mandarina
Source: PeerJ. 2021 Feb 3;9:e10818. doi: 10.7717/peerj.10818 (PMC7866900; doi:10.7717/peerj.10818)
Supplement: Supplemental Information 1 [file peerj-09-10818-s001.docx]

# Table S1

Information for species used in this paper.

| Latin name | Data source and Accession number or URL |
| --- | --- |
| *Amyelois transitella* | NCBI RefSeq Accession: GCF_001186105.1 |
| *Bicyclus anynana* | NCBI RefSeq Accession: GCF_900239965.1 |
| *Chilo suppressalis* | NCBI GenBank GCA_004000445.1 |
| *Danaus plexippus* | NCBI GenBank GCA_000235995.2 |
| *Galleria mellonella* | NCBI RefSeq Accession: GCF_003640425.1 |
| *Heliconius melpomene* | Ensembl Metazoa release 43 |
| *Helicoverpa armigera* | NCBI RefSeq Accession: GCF_002156985.1 |
| *Heliothis virescens* | NCBI GenBank GCA_002382865.1 |
| *Hyposmocoma kahamanoa* | NCBI RefSeq Accession: GCF_003589595.1 |
| *Melitaea cinxia* | Ensembl Metazoa release 43 |
| *Operophtera brumata* | NCBI GenBank GCA_001266575.1 |
| *Ostrinia furnacalis* | NCBI RefSeq Accession: GCF_004193835.1 |
| *Papilio machaon* | NCBI RefSeq Accession: GCF_001298355.1 |
| *Papilio polytes* | NCBI RefSeq Accession: GCF_000836215.1 |
| *Papilio xuthus* | NCBI RefSeq Accession: GCF_000836235.1 |
| *Pieris rapae* | NCBI RefSeq Accession: GCF_001856805.1 |
| *Plutella xylostella* | NCBI RefSeq Accession: GCF_000330985.1 |
| *Spodoptera litura* | NCBI RefSeq Accession: GCF_002706865.1 |
| *Trichoplusia ni* | NCBI RefSeq Accession: GCF_003590095.1 |
| *Vanessa tameamea* | NCBI RefSeq Accession: GCF_002938995.1 |
| *Bombyx mandarina* | NCBI RefSeq Accession: GCF_003987935.1 |
| *Bombyx mori* | <http://silkbase.ab.a.u-tokyo.ac.jp/cgi-bin/download.cgi> |

# Table S2

The chromosome length of each chromosome in the wild and domestic silkworms. Size variation is defined as |log10(wild/domestic)| and (wild-domestic)/domestic, where ‘wild’ is the chromosome length of wild silkworm and ‘domestic’ is the homologous chromosome length of domestic silkworm.

| Chromosome | Wild | | Domestic | size variation | |
| --- | --- | --- | --- | --- | --- |
|  | Length | #scaffolds | Length | \|log10(wild/domestic)\| | (wild-domestic)/  domestic |
| 1 | 21,193,164 | 8 | 20,666,287 | 0.01 | 0.03 |
| 2 | 7,258,460 | 6 | 8,396,445 | 0.06 | -0.14 |
| 3 | 12,267,409 | 12 | 15,212,953 | 0.09 | -0.19 |
| 4 | 9,438,841 | 20 | 18,737,234 | 0.30 | -0.50 |
| 5 | 12,572,746 | 18 | 19,061,979 | 0.18 | -0.34 |
| 6 | 8,529,492 | 9 | 16,650,604 | 0.29 | -0.49 |
| 7 | 11,008,558 | 14 | 13,944,894 | 0.10 | -0.21 |
| 8 | 16,756,911 | 17 | 16,262,221 | 0.01 | 0.03 |
| 9 | 17,957,567 | 7 | 16,796,068 | 0.03 | 0.07 |
| 10 | 10,891,496 | 16 | 17,614,771 | 0.21 | -0.38 |
| 11 | 15,302,606 | 15 | 20,440,007 | 0.13 | -0.25 |
| 12 | 17,428,063 | 19 | 17,580,608 | 0.00 | -0.01 |
| 13 | 14,397,155 | 12 | 17,735,081 | 0.09 | -0.19 |
| 14 | 20,652,042 | 7 | 13,345,518 | 0.19 | 0.55 |
| 15 | 15,086,344 | 14 | 18,440,292 | 0.09 | -0.18 |
| 16 | 11,012,451 | 12 | 14,337,292 | 0.11 | -0.23 |
| 17 | 18,736,880 | 8 | 16,840,672 | 0.05 | 0.11 |
| 18 | 12,619,989 | 14 | 15,699,053 | 0.09 | -0.20 |
| 19 | 18,379,601 | 5 | 14,801,489 | 0.09 | 0.24 |
| 20 | 10,922,641 | 13 | 12,370,531 | 0.05 | -0.12 |
| 21 | 8,279,021 | 7 | 15,310,392 | 0.27 | -0.46 |
| 22 | 10,416,726 | 13 | 18,482,526 | 0.25 | -0.44 |
| 23 | 16,898,563 | 26 | 21,465,692 | 0.10 | -0.21 |
| 24 | 22,989,029 | 15 | 17,359,173 | 0.12 | 0.32 |
| 25 | 13,001,450 | 14 | 14,548,897 | 0.05 | -0.11 |
| 26 | 14,682,749 | 6 | 11,473,476 | 0.11 | 0.28 |
| 27 | 12,904,033 | 4 | 10,930,128 | 0.07 | 0.18 |
| 28 | 10,450,270 | 6 | 10,609,739 | 0.01 | -0.02 |
| Total | 392,034,257 |  | 445,114,022 |  |  |

# Table S3

Statistics for raw transcriptome sequencing data from published papers. Bmor: *Bombyx mori*, Bman: *Bombyx mandarina*. ‘*’ means that, for the sample, if the number of raw reads is too large, we sampled only approximately 30 million read pairs.

| **Species** | Tissue | Sex | Abbr. | **Ref.** | **SRR** | Raw | | Mapping rate |
| --- | --- | --- | --- | --- | --- | --- | --- | --- |
|  |  |  |  |  |  | Reads | Used Reads* |  |
| Bman | middle silk glands | Male | MSG | [[1](#_ENREF_1)] | SRR1619431 | 62,612,070 | 62,612,070 | 81.73% |
| Bman | posterior silk glands | Male | PSG | [[1](#_ENREF_1)] | SRR1619469 | 57,769,130 | 57,769,130 | 69.65% |
| Bmor | anterior middle silkgland | Male | AMSG_M | [[2](#_ENREF_2)] | SRR4425252 | 225,900,902 | 79,188,889 | 95.33% |
| Bmor | anterior middle silkgland | Female | AMSG_F | [[2](#_ENREF_2)] | SRR4425253 | 252,567,550 | 78,132,032 | 94.90% |
| Bmor | anterior silkgland | Male | ASG_M | [[2](#_ENREF_2)] | SRR4425254 | 177,969,128 | 67,662,864 | 94.78% |
| Bmor | anterior silkgland | Female | ASG_F | [[2](#_ENREF_2)] | SRR4425255 | 212,475,030 | 66,516,702 | 95.46% |
| Bmor | posterior silkgland | Male | PSG_M | [[2](#_ENREF_2)] | SRR4425260 | 201,976,336 | 85,905,644 | 91.36% |
| Bmor | posterior silkgland | Female | PSG_F | [[2](#_ENREF_2)] | SRR4425261 | 288,000,000 | 83,406,725 | 94.02% |
| Bmor | posterior middle silkgland | Male | PMSG_M | [[2](#_ENREF_2)] | SRR4425256 | 222,914,024 | 86,827,392 | 93.94% |
| Bmor | posterior middle silkgland | Female | PMSG_F | [[2](#_ENREF_2)] | SRR4425257 | 267,951,558 | 77,027,864 | 93.21% |
| Bmor | middle of middle silkgland | Male | MMSG_M | [[2](#_ENREF_2)] | SRR4425258 | 226,080,784 | 79,745,830 | 95.29% |
| Bmor | middle of middle silkgland | Female | MMSG_F | [[2](#_ENREF_2)] | SRR4425259 | 368,215,760 | 70,945,656 | 95.29% |

# Table S4

Functional annotation of proteome from the wild and domestic silkworm. ‘Annotated’ is the number of genes annotated by at least one following four functional databases.

| Database | Wild silkworm | | Domestic silkworm | |
| --- | --- | --- | --- | --- |
|  | #genes | percent | #genes | percent |
| Nr | 13,100 | 92.18% | 15,033 | 89.06% |
| SwissProt | 7,195 | 50.63% | 8,199 | 48.57% |
| KEGG | 6,469 | 45.52% | 7,572 | 44.86% |
| GO | 8,244 | 58.01% | 9,546 | 56.55% |
| Annotated | 13,413 | 94.38% | 16,060 | 95.14% |
| Non-annotated | 799 | 5.62% | 820 | 4.86% |
| total | 14,212 |  | 16,880 |  |

# Table S5

The total number of cytochrome P450 genes identified in each species.

| Species | # high quality  genes | # cytochrome  P450 genes |
| --- | --- | --- |
| *Amyelois transitella* | 13,933 | 96 |
| *Bicyclus anynana* | 14,119 | 114 |
| *Chilo suppressalis* | 15,653 | 73 |
| *Danaus plexippus* | 14,993 | 78 |
| *Galleria mellonella* | 13,686 | 102 |
| *Heliconius melpomene* | 12,627 | 114 |
| *Helicoverpa armigera* | 13,525 | 101 |
| *Heliothis virescens* | 14,965 | 98 |
| *Hyposmocoma kahamanoa* | 13,798 | 89 |
| *Melitaea cinxia* | 16,558 | 81 |
| *Operophtera brumata* | 16,903 | 125 |
| *Ostrinia furnacalis* | 14,930 | 87 |
| *Papilio machaon* | 13,620 | 101 |
| *Papilio polytes* | 11,933 | 112 |
| *Papilio xuthus* | 14,860 | 88 |
| *Pieris rapae* | 12,268 | 83 |
| *Plutella xylostella* | 17,763 | 97 |
| *Spodoptera litura* | 15,705 | 121 |
| *Trichoplusia ni* | 14,615 | 110 |
| *Vanessa tameamea* | 11,721 | 64 |
| *Bombyx mandarina* | 14,212 | 68 |
| *Bombyx mori* | 16,880 | 83[[3](#_ENREF_3)] |

# Table S6

Significantly enriched GO terms of the wild silkworm cytochrome P450 genes.

| GO | Class | #genes | P value | Term |
| --- | --- | --- | --- | --- |
| GO:0005506 | MF | 91 | 0 | iron ion binding |
| GO:0020037 | MF | 107 | 0 | heme binding |
| GO:0016705 | MF | 109 | 0 | oxidoreductase activity, acting on paired donors, with  incorporation or reduction of molecular oxygen |
| GO:0004497 | MF | 16 | 0.00039 | monooxygenase activity |
| GO:0055114 | BP | 424 | 0 | oxidation-reduction process |

# Table S7

KEGG annotation of the wild silkworm cytochrome P450 genes. Genes with green color involves in the KEGG map00981 (Insect hormone biosynthesis).

| KO | Description | GeneID |
| --- | --- | --- |
| K07427 | docosahexaenoic acid omega-hydroxylase [EC:1.14.14.79] | MSTRG.17906.1 |
| K07427 | docosahexaenoic acid omega-hydroxylase [EC:1.14.14.79] | XP_028026371.1 |
| K07427 | docosahexaenoic acid omega-hydroxylase [EC:1.14.14.79] | XP_028043540.1 |
| K07427 | docosahexaenoic acid omega-hydroxylase [EC:1.14.14.79] | XP_028043541.1 |
| K07427 | docosahexaenoic acid omega-hydroxylase [EC:1.14.14.79] | XP_028043542.1 |
| K10720 | ecdysteroid 25-hydroxylase | XP_028040956.1 |
| K10721 | ecdysteroid 22-hydroxylase | XP_028032374.1 |
| K10722 | ecdysteroid 2-hydroxylase | XP_028033300.1 |
| K10723 | ecdysone 20-monooxygenase [EC:1.14.99.22] | XP_028030999.1 |
| K14937 | farnesoate epoxidase [EC:1.14.14.127 1.14.14.128] | XP_028039023.1 |
| K14939 | cytochrome P450 family 307 subfamily A | XP_028041590.1 |
| K14985 | 26-hydroxylase [EC:1.14.-.-] | XP_028040961.1 |
| K14985 | 26-hydroxylase [EC:1.14.-.-] | XP_028040963.1 |
| K14999 | cytochrome P450 family 6 [EC:1.14.-.-] | MSTRG.11650.2 |
| K14999 | cytochrome P450 family 6 [EC:1.14.-.-] | MSTRG.12374.1 |
| K14999 | cytochrome P450 family 6 [EC:1.14.-.-] | XP_028026744.1 |
| K14999 | cytochrome P450 family 6 [EC:1.14.-.-] | XP_028026755.1 |
| K14999 | cytochrome P450 family 6 [EC:1.14.-.-] | XP_028026771.1 |
| K14999 | cytochrome P450 family 6 [EC:1.14.-.-] | XP_028026790.1 |
| K14999 | cytochrome P450 family 6 [EC:1.14.-.-] | XP_028026792.1 |
| K14999 | cytochrome P450 family 6 [EC:1.14.-.-] | XP_028026857.1 |
| K14999 | cytochrome P450 family 6 [EC:1.14.-.-] | XP_028026883.1 |
| K14999 | cytochrome P450 family 6 [EC:1.14.-.-] | XP_028027733.1 |
| K14999 | cytochrome P450 family 6 [EC:1.14.-.-] | XP_028030266.1 |
| K14999 | cytochrome P450 family 6 [EC:1.14.-.-] | XP_028030488.1 |
| K14999 | cytochrome P450 family 6 [EC:1.14.-.-] | XP_028033575.1 |
| K14999 | cytochrome P450 family 6 [EC:1.14.-.-] | XP_028035792.1 |
| K14999 | cytochrome P450 family 6 [EC:1.14.-.-] | XP_028037108.1 |
| K14999 | cytochrome P450 family 6 [EC:1.14.-.-] | XP_028037243.1 |
| K14999 | cytochrome P450 family 6 [EC:1.14.-.-] | XP_028037754.1 |
| K14999 | cytochrome P450 family 6 [EC:1.14.-.-] | XP_028039890.1 |
| K14999 | cytochrome P450 family 6 [EC:1.14.-.-] | XP_028039903.1 |
| K14999 | cytochrome P450 family 6 [EC:1.14.-.-] | XP_028040226.1 |
| K15001 | cytochrome P450 family 4 [EC:1.14.-.-] | XP_028028458.1 |
| K15001 | cytochrome P450 family 4 [EC:1.14.-.-] | XP_028031815.1 |
| K15001 | cytochrome P450 family 4 [EC:1.14.-.-] | XP_028033176.1 |
| K15001 | cytochrome P450 family 4 [EC:1.14.-.-] | XP_028033210.1 |
| K15001 | cytochrome P450 family 4 [EC:1.14.-.-] | XP_028036358.1 |
| K15001 | cytochrome P450 family 4 [EC:1.14.-.-] | XP_028036359.1 |
| K15001 | cytochrome P450 family 4 [EC:1.14.-.-] | XP_028036360.1 |
| K15001 | cytochrome P450 family 4 [EC:1.14.-.-] | XP_028036361.1 |
| K15001 | cytochrome P450 family 4 [EC:1.14.-.-] | XP_028036416.1 |
| K15001 | cytochrome P450 family 4 [EC:1.14.-.-] | XP_028036546.1 |
| K15001 | cytochrome P450 family 4 [EC:1.14.-.-] | XP_028040208.1 |
| K15001 | cytochrome P450 family 4 [EC:1.14.-.-] | XP_028040566.1 |
| K15001 | cytochrome P450 family 4 [EC:1.14.-.-] | XP_028040567.1 |
| K15001 | cytochrome P450 family 4 [EC:1.14.-.-] | XP_028040570.1 |
| K15001 | cytochrome P450 family 4 [EC:1.14.-.-] | XP_028040572.1 |
| K15001 | cytochrome P450 family 4 [EC:1.14.-.-] | XP_028040841.1 |
| K15001 | cytochrome P450 family 4 [EC:1.14.-.-] | XP_028041506.1 |
| K15001 | cytochrome P450 family 4 [EC:1.14.-.-] | XP_028042103.1 |
| K15001 | cytochrome P450 family 4 [EC:1.14.-.-] | XP_028042717.1 |
| K15003 | cytochrome P450 family 9 [EC:1.14.-.-] | XP_028033856.1 |
| K15003 | cytochrome P450 family 9 [EC:1.14.-.-] | XP_028035894.1 |
| K15003 | cytochrome P450 family 9 [EC:1.14.-.-] | XP_028036963.1 |
| K15003 | cytochrome P450 family 9 [EC:1.14.-.-] | XP_028036964.1 |
| K15003 | cytochrome P450 family 9 [EC:1.14.-.-] | XP_028036982.1 |
| K15003 | cytochrome P450 family 9 [EC:1.14.-.-] | XP_028036989.1 |
| K15003 | cytochrome P450 family 9 [EC:1.14.-.-] | XP_028042624.1 |
| K15004 | cytochrome P450 family 12 [EC:1.14.-.-] | XP_028028461.1 |
| K15004 | cytochrome P450 family 12 [EC:1.14.-.-] | XP_028028513.1 |
| K15004 | cytochrome P450 family 12 [EC:1.14.-.-] | XP_028042905.1 |
| K17960 | cytochrome P450 family 49 subfamily A | XP_028042777.1 |

# Figure S1

The phylogenetic tree and characteristics of the cytochrome P450 genes in the wild silkworm. For visualization purpose, the tree was rooted at the node separating the mitochondrial cytochrome P450s clan to other clans. The first column next to the tree assigns each protein to clans. The next column is the exon-intron organization, and the horizontal lines represent the gene regions and the vertical bars indicate exon locations. The third column shows the positions of conserved P450 domain in the P450 proteins.


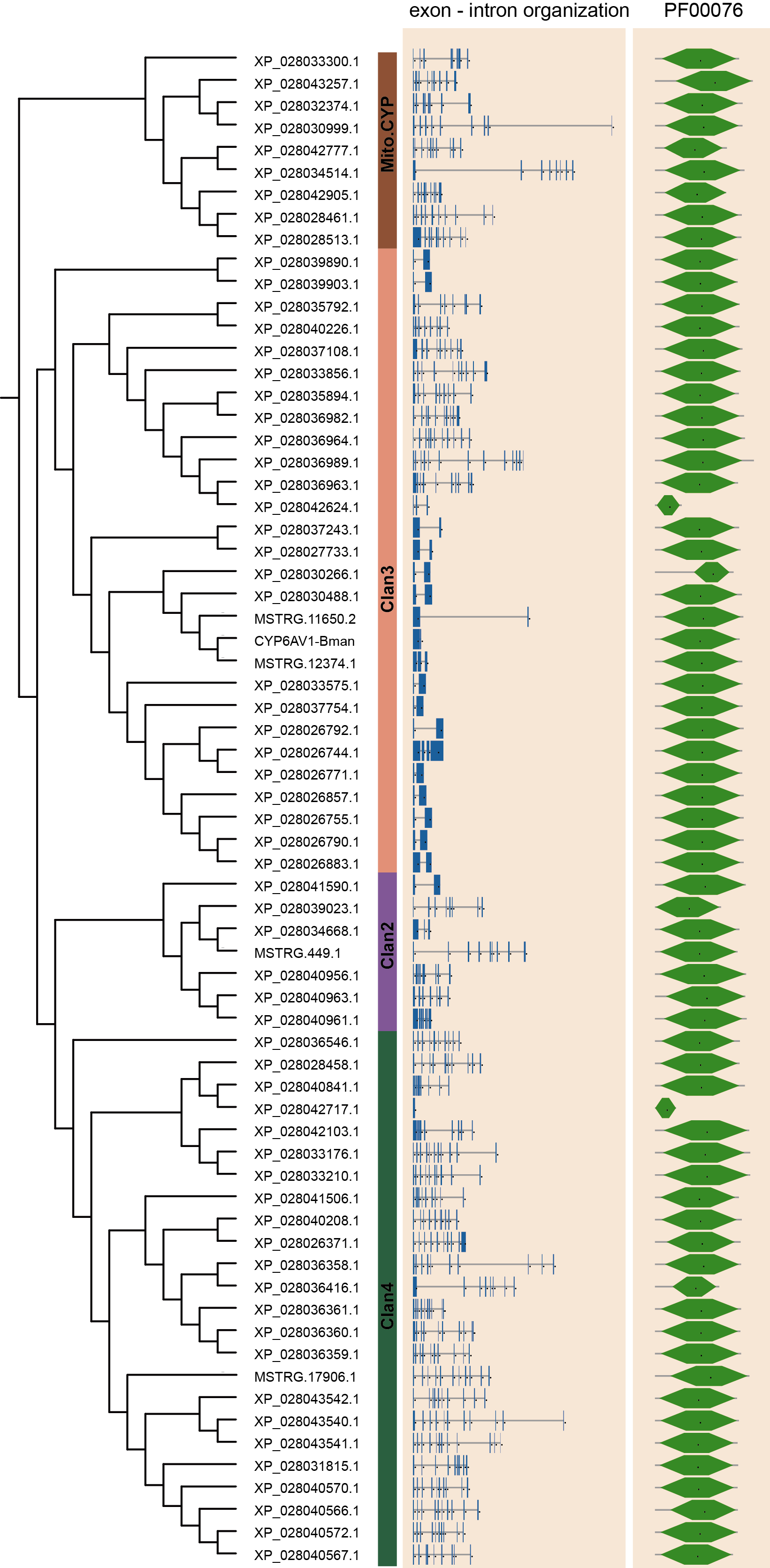


**References**

1. Cheng, T.; Fu, B.; Wu, Y.; Long, R.; Liu, C.; Xia, Q., Transcriptome sequencing and positive selected genes analysis of Bombyx mandarina. In *PLoS One*, 2015; Vol. 10, p e0122837.

2. Chang, H.; Cheng, T.; Wu, Y.; Hu, W.; Long, R.; Liu, C.; Zhao, P.; Xia, Q., Transcriptomic Analysis of the Anterior Silk Gland in the Domestic Silkworm (Bombyx mori) – Insight into the Mechanism of Silk Formation and Spinning. *PLoS One* **2015,** 10, (9), e0139424.

3. Kawamoto, M.; Jouraku, A.; Toyoda, A.; Yokoi, K.; Minakuchi, Y.; Katsuma, S.; Fujiyama, A.; Kiuchi, T.; Yamamoto, K.; Shimada, T., High-quality genome assembly of the silkworm, Bombyx mori. *Insect Biochem Mol Biol* **2019,** 107, 53-62.
